# Compliance Indicators of COVID-19 Prevention and Vaccines Hesitancy in Kenya: A Random-Effects Endogenous Probit Model

**DOI:** 10.3390/vaccines9111359

**Published:** 2021-11-19

**Authors:** Abayomi Samuel Oyekale

**Affiliations:** Department of Agricultural Economics and Extension, North-West University Mafikeng Campus, Mmabatho 2735, South Africa; abayomi.oyekale@nwu.ac.za

**Keywords:** compliance indicator, COVID-19, vaccine hesitancy, random-effects, probit model, endogenous, Kenya

## Abstract

Vaccine hesitancy remains a major public health concern in the effort towards addressing the COVID-19 pandemic. This study analyzed the effects of indicators of compliance with preventive practices on the willingness to take COVID-19 vaccines in Kenya. The data were from the COVID-19 Rapid Response Phone Surveys conducted between January and June 2021 during the fourth and fifth waves. The data were analyzed with the random-effects endogenous Probit regression model, with estimated parameters tested for robustness and stability. The results showed that willingness to take vaccines increased between the fourth and fifth waves. Compliance with many of the preventive practices also improved, although the utilizations of immune system-promoting practices were very low. The panel Probit regression results showed that compliance indicators were truly endogenous and there was existence of random effects. Immune system-boosting and contact-prevention indicators significantly increased and decreased the willingness to take vaccines, respectively (*p* < 0.01). The experience of mental health disorders in the form of nervousness and hopelessness also significantly influenced vaccine hesitancy (*p* < 0.10). Willingness to take vaccines also significantly increased among older people and those with a formal education (*p* < 0.01). Different forms of association exist between vaccine hesitancy and the prevention compliance indicators. There is a need to properly sensitize the people to the need to complement compliance with COVID-19 contact-prevention indicators with vaccination. Addressing mental health disorders in the form of loneliness, nervousness, depression, hopelessness and anxiety should also become the focus of public health, while efforts to reduce vaccine hesitancy should focus on individuals without formal education, males and youths.

## 1. Introduction

COVID-19 came into the limelight of health policy discourse in Kenya on 13 March 2021, when the first positive case was announced by the Ministry of Health [1]. As of 27 October 2021, there have been 252,938 positive cases out of the 2,682,247 tests that were conducted and 5266 deaths [2]. These statistics further reveal a case–fatality ratio (CFR) of 2.08%, which is a bit lower than the 2.57% CFR for Africa as a whole [3]. Some counties in Kenya have gone through some mandatory lockdowns [4,5], which disrupted several economic activities [6,7]. The country has also gone through four infection spikes [8,9,10] even though the government is using every means to enforce compliance with some preventive practices [11,12]. These include the wearing of face masks, avoidance of crowds, the use of hand sanitizers and social distancing [4,13]. People are also being advised to adopt healthy lifestyles, such as reductions in stress levels, regular exercise, the consumption of balanced diets and the intake of certain antioxidant-loaded foods such as fruits, vegetables, culinary herbs and some spices for the enhancement of the functionality of the immune system [14]. This is because healthy lifestyles are part of the essential prerequisites for living and maintaining a strong and healthy life. Weakness of the immune system compromises the ability of the body to release some essential antibodies in the fight against infections, thereby promoting a susceptibility to some forms of severe or chronic pathogenic infections [14,15].

Moreover, advocating for adequate nutrition is a welcome development in the fight against the COVID-19 pandemic [16,17]. However, with or without pandemics, the role of adequate nutrition in promoting sound health cannot be overemphasized [18]. Specifically, sound immune systems have been found to facilitate the fight against some previous hybrids of coronaviruses [19]. Foods that are rich in vitamins generally have some antioxidant properties and they render some immunomodulatory benefits [20]. It has been noted that COVID-19, being a respiratory tract infection, can be prevented with sufficient intake of vitamin D [21], while vitamins C and E are able to subdue free radicals through their antioxidant properties [22]. Although the responses from the body’s immune system to some specific infections differ depending on the nature of the infecting agents, the viral load, infection route, age, genetic composition, comorbidity and previous exposure [23], the magnitude of resulting tissue damages can be significantly reduced depending on the degree of the innate and adaptive immune system, which is also reckoned as a critical determinant of the outcome of administered clinical treatments [24]. Therefore, proper nutrition can reduce the severity of infection in some patients with a COVID-19 infection. Some studies have highlighted the anti-inflammatory properties of niacin [25], the effectiveness of folic acid [26], the therapeutic properties of niacin [27,28], the regulatory function of vitamin B in the formation of chemokine/cytokine and arbitrate [29,30] and the functions of vitamin C in combatting sepsis and acute respiratory distress syndrome (ARDS) [31] in successful treatments of COVID-19.

In addition to nutrition, healthcare practitioners are now emphasizing the need to promote mass vaccination as a way of strengthening individuals’ immune systems [32]. This is also going to ensure the achievement of herd immunity. However, with the expectation that herd immunity will be achieved with 80% full vaccination coverage [33], it is unclear how long it will take Kenya to meet this requirement, given that, as of 26 October 2021, only 5.40% of the Kenyan adult population has been fully vaccinated [2]. Although vaccination is an integral component of public health service-delivery and management in Kenya [34], management of COVID-19 through vaccination generally portends some serious concerns [35]. These emanate from some social-media-circulated misinformation on the efficacy and side effects of some administered vaccines [36].

The subject of vaccine hesitancy is of global public health relevance in the context of COVID-19 management, and there are numerous socioeconomic and demographic factors that influence individuals’ decisions [37]. COVID-19 vaccine hesitancy is largely emanating from some confirmed side effects and unfounded rumors of vaccine-related deaths in some countries [38,39,40,41]. This development negates the achievement of the resolution of May 2020 at the 73rd World Health Assembly, where mass vaccination was identified as a prerequisite for the timely containment of COVID-19 [42]. Moreover, it has been emphasized that inadequate coverage of vaccines in a particular country could undermine global efforts at addressing the ongoing pandemic due to the mutation tendency of the virus [43,44,45].

Although some studies have indicated the role of gender, age and education in explaining individuals’ compliance with COVID-19 preventive methods [29,30,31], the linkage between indicators of compliance with preventive methods and vaccine hesitancy is not well-studied in the literature. Some other studies have reported that noncompliance with COVID-19 preventive methods is linked with some negative attitudes, perception of associated health and general welfare risks, convictions on the existence of the virus, the existence of penalties for non-compliant persons, affordability and access to protective materials and the possibility or ease of working remotely [46,47,48]. Some studies have analyzed the effect of demographic and socioeconomic factors on vaccine hesitancy with mixed results [49,50,51,52,53,54,55,56,57,58,59,60,61]. Others have emphasized assessments of health risk [62,63]. Such risk is evaluated based on the understanding of an individual’s susceptibility to being infected, the severity of morbidity and the likelihood of fatality [64,65,66,67,68,69,70,71].

This study seeks to analyze the effect of COVID-19 preventive compliance indicators on vaccine hesitancy. The first hypothesis states that the contact-prevention compliance indicator does not significantly influence vaccine hesitancy. In the second hypothesis, it is stated that the immune system-boosting compliance indicator does not significantly influence vaccine hesitancy. The study seeks to add to the existing body of knowledge in some major ways. First, there is a dearth of studies on the linkage between indicators of COVID-19 protective compliance and vaccine hesitancy. Understanding such linkages can assist public health policy makers in formulating effective mechanisms for ensuring adequate protection of the entire people against COVID-19. Second, the study is adding some empirical strength for analyzing vaccine hesitancy through the utilization of panel data. Specifically, the estimation of a random-effects COVID-19 vaccine hesitancy model is very rare in the literature due to a paucity of required data. Therefore, factoring individuals’ heterogenous characteristics into an understanding willingness to get COVID-19 vaccines can reflect the inherent changes across time that can have significant relevance to public health policy. This study will therefore serve as a veritable source of information for informed public health decision making, given the ongoing vaccine hesitancy in many developing and developed countries.

## 2. Materials and Methods

### 2.1. Data and Sampling Procedures

This study used Kenya’s COVID-19 Rapid Response Phone Survey that was conducted in 2020 and 2021 [72]. The data collection was conducted by the Kenya National Bureau of Statistics and the University of California, Berkeley, with support from the World Bank. The study was ethically reviewed and approved by the Ethical Committee of the Kenya National Bureau of Statistics. The surveys sought to track COVID-19 compliance with preventive protocols and the socioeconomic impacts of the pandemic on Kenyan households. The survey used the sampling frame of the 2015/2016 Kenya Integrated Household Budget Survey (KIHBS), which was a Computer-Assisted Personal Interviewing (CAPI) pilot survey that also collected the respondents’ phone numbers. The modus operandi of the survey was to conduct a longitudinal survey, whereby selected households were to be interviewed once every two months.

The sampling proceeded by forming a sampling frame with the 9007 households that presented valid phone numbers from the 2015/2016 KIHBS, and introductory text messages were sent to 5000 randomly selected phone numbers to confirm if they were still in use. It was found that 4075 of those numbers were still active. These individuals formed the primary sampling frame for the baseline survey. The selected phone numbers were then called, and the respondents had to be 18 years of age or older to be eligible for the interview. The respondents’ consents were obtained and an objection to granting participation consent automatically brought the interview to an end. Interviews were conducted in the respondent’s best understood language since the questionnaire was translated into 12 other languages, which were “Swahili, Luo, Arabic, French, Kirundi, Luganda, Oromo, Somali, Kinyarwanda, Tigrinya, Nuer, and Dinka” [73]. The questionnaire was comprised of different sections, including type of employment, income generating activities, coping strategies against COVID, experience of food insecurity, access to education facilities and health services, perception of subjective wellbeing, knowledge of COVID-19, behavior changes in response to the pandemic and perceptions of effectiveness of the government’s responses to the pandemic. The questionnaire was pretested among a smaller group of people, after which some corrections were made to ensure the simplification of some words [7].

The data were collected by enumerators through phone calls. If the answering persons granted the consent to participate, the data were collected and a compensation of 50 shillings was given after the completion of the interview. More specifically, an adult member in the household was spoken to if the targeted respondent is not available. The survey used CAPI, which is a phone survey in which data are captured by tablets and mobile phones. Captured data are transferred to a central server, which can only be accessed by authorized people with approved usernames and passwords.

Since the commencement of the surveys in May 2020, data for five waves had been collected. The first wave covered 4063 households in a survey that was conducted between 14 May and 7 July 2020. The second wave was comprised of 4504 households and it was conducted between 16 July and 18 September 2020. The third wave was comprised of 4993 households and it was implemented between 18 September and 28 November 2020. Additionally, 4860 households completed the fourth wave of the survey that was implemented between 15 January and 25 March 2021. Finally, in the fifth wave, 5854 households were interviewed between 29 March and 13 June 2021. This study utilized the data for the fourth and fifth waves because they contained information on COVID-19 vaccine hesitancy. However, because of missing data in some of the variables, the data that were utilized for this study comprised of 4867 and 5835 respondents in waves 4 and 5, respectively.

### 2.2. Estimated Models

This study estimated a random-effects Probit regression model with endogenous regressors. The parameters were estimated with a maximization of the likelihood function through the maximum likelihood estimators. The indicators of compliance with COVID-19 preventive methods, which are part of the explanatory variables, were suspected to be endogenous. These indicators were computed with Principal Component Analysis (PCA) using the questions that were asked on behaviour change. These questions were divided into two parts. Part 1 comprised those behaviours that are related to prevention of contacts or spreading of the COVID-19 virus which respondents observed in the past 7 days. These were contained in section 10 and question 13 of the questionnaire. These practices were hand-washing, no hand-shaking, avoidance of groups of more than 10 people, hand-sanitization, covering the mouth when coughing, staying home, traveling less, working less, wearing masks and stocking food at home. The second part comprised immune system-boosting behaviours, which are drinking tea with lemon, drinking warm water, the consumption of vitamin C rich fruits, eating garlic and fruits such as avocadoes and mangoes, eating alkaline food and drinking bicarbonate. The construction of these indices presented some advantages. First, it helped to reduce the number of the variables to one which was quite manageable. The second is that it helped with estimating the model without having to deal with the serious problem of multicollinearity among these variables, since some behaviour-change attribute variables may be highly correlated. More importantly, PCA is a statistical method that reduces several data variables into a composite index by utilizing the basic statistical information within the main variables [73,74].

The model specification begins with an estimation of a panel Probit regression with two suspected endogenous regressors:

(1) $$Y_{it}=\overset{n}{\underset{k=1}{\sum }}\beta_{k}X_{it}+\theta C_{it}+\omega Z_{it}+v_{t}+\epsilon_{it}$$

The equations for the endogenous regressors are specified as:

(2) $$C_{it}=\kappa +\overset{n}{\underset{k=1}{\sum }}\varphi_{k}X_{it}+\overset{2}{\underset{d=1}{\sum }}\delta_{d}I_{it}+e_{it}$$

(3) $$Z_{it}=\gamma +\overset{n}{\underset{k=1}{\sum }}\eta_{k}X_{it}+\overset{2}{\underset{d=1}{\sum }}\alpha_{d}I_{it}+m_{it}$$

where *i* denotes individual respondents, t is the time subscript of the panel data and $v_{t}$ represents the random effects of the panel specification. $Y_{it}$ is the dependent variable that was coded as 1 for a willingness to take COVID-19 vaccines and 0 if otherwise. The endogenous regressors $C_{it}$ and $Z_{it}$ are the composite indicators of compliance with respect to COVID-19 virus contact-avoidance and immune system-boosting behaviour, respectively. In addition, $\beta_{k},$ $\varphi_{k}$, $\eta_{k}$ θ, δ, α, ω, κ and γ are the estimated parameters, and $X_{it}$ are the explanatory variables (see Table 1). $I_{it}$ denotes instrumental variables which are: feeling nervous due to COVID-19 (yes = 1, 0 otherwise) and the number of days experiencing depression in a week. These instruments are expected to be highly correlated with the endogenous regressors but uncorrelated with a willingness to take vaccines. Additionally, $\epsilon_{it}$, $e_{it}$ and $m_{it}\;$ are the error components of the models. To estimate consistent parameters for Equation (1) in the presence of $C_{it}$ and $Z_{it}$ that are suspected to be endogenous, an instrumental variable Probit regression approach was used. Therefore, the xteprobit command of STATA 17 software was to be invoked. This command implements a random-effects Probit regression model with endogenous explanatory variables [75]. However, given the number of explanatory variables, the xteprobit command takes quite a long time to run. Therefore, in this study, an alternative way of correcting the endogeneity was used. This involved invoking the conventional xteprobit command for estimating random-effects Probit models with the inclusion of the error terms $e_{it}$ and $m_{it}$ that were generated in Equations (2) and (3), respectively, as part of the independent variables. If the estimated parameters for $e_{it}$ and $m_{it}$ are not statistically significant (*p* > 0.05), endogeneity is not present. Therefore, the estimated equation is specified as:

(4) $$Y_{it}=\overset{n}{\underset{k=1}{\sum }}\beta_{k}X_{it}+\theta C_{it}+\omega Z_{it}+\psi e_{it}+\tau m_{it}+v_{t}+\epsilon_{it\;\;\;\;\;\;\;\;\;\;\;\;\;\;\;\;\;\;\;\;}$$

Therefore, if ψ  and τ show statistical significance (*p* < 0.05), it implies that $C_{it}\;$ is truly endogenous and the error term in Equation (1) is correlated with the indicators of compliance ($C_{it}\;$ and $Z_{it}$). In addition, estimating the appropriateness of the random-effects model in Equation (4) requires testing for the statistical significance of the computed rho in STATA software, after estimating the parameters of the variables in Equation (4) with the xteprobit command. The value of rho, which is the proportion of the total variance that had been contributed by the panel level variance component, was provided by STATA software. The software also provides a likelihood-ratio test statistic for rho being equal to zero (*p* < 0.05). This test seeks to confirm the appropriateness of using a random-effects model. If the null hypothesis (rho = 0) is accepted, estimating a standard Probit model would produce the same result as the one obtained from a panel Probit regression. This also implies the complete absence of any form of heterogeneity across the periods of the panel data.

The stability and robustness of the estimated variables were tested because the command was invoked using a quadrature approach, with its parameter accuracy depending on the number of integration points [75]. The quadchk command was therefore invoked to confirm the accuracy of the estimated parameters by comparing the results that were obtained with other different integration points. The intention here was to look out for relative differences in the estimated parameters that were more than 1 percent [75]. If a significant difference exists, the parameter cannot be interpreted. In this study, very insignificant differences were found between the two results. This is a confirmation of the robustness and stability of the estimated parameters. The Variance Inflation Factor (VIF) was also used to test for multicollinearity among the included explanatory variables. Setting a cutoff point for the range of acceptable values of VIF is sometimes controversial [76], although a value above 10 is considered significantly worrisome. Additionally, the Breuch-Pagan test was conducted to test for heteroscedasticity in the estimated models for the determinants of compliance indicators (Equations (2) and (3)). If this test shows statistical significance, there is the presence of heteroscedasticity and the model should be estimated with a robust standard error [76].

## 3. Results

### 3.1. Selected Demographic Characteristics of the Respondents

**Figure 1:**
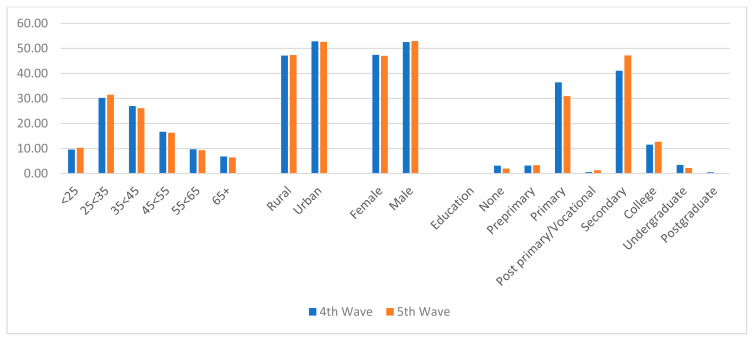
Distribution of respondents’ selected demographic variables.

Figure 1 shows the distribution of selected demographic characteristics of the respondents. It reveals that during waves 4 and 5, more than half of the respondents were between 25 and 44 years of age. Rural households constituted a lower proportion of the respondents, with 47.15% and 47.37% in waves 4 and 5, respectively. Male respondents also constituted higher percentages, with 52.56% and 52.96% in waves 4 and 5, respectively. Figure 1 further shows that the majority of the respondents were either holders of primary or secondary school certificates.

### 3.2. Compliance with COVID-19 Preventive Behaviours and Computed Indicators

Table 2 shows the results of compliance with recommended COVID-19 preventive and immune system-boosting behaviours during the fourth and fifth waves of the panel surveys. It reveals that between wave 4 and wave 5, the percentage of the people that were washing hands more regularly increased from 89.30% to 93.11%. The proportion of the respondents that were avoiding groups of more than 10 persons increased from 70.68% in wave 4 to 81.68% in wave 5. Other behaviour-change indicators, such as the avoidance of handshakes, the wearing of face masks and the use of hand sanitizers also showed some increases between waves 4 and 5. However, between waves 4 and 5, there were some reductions in the proportion of the respondents that were covering their mouths when coughing, staying at home, travelling less, working less and stocking up food. On the aspect of some behaviours that can boost the immune system, between waves 4 and 5, those respondents that were drinking tea with lemons decreased from 8.30% to 5.47%. However, those that were eating garlic and fruits (lemons, mangoes, avocadoes) increased from 2.96% during wave 4 to 7.88% during wave 5, respectively. Consumption of fruits that are rich in vitamin C decreased from 2.75% to 2.06% during wave 4 and wave 5, respectively.

**Figure 2:**
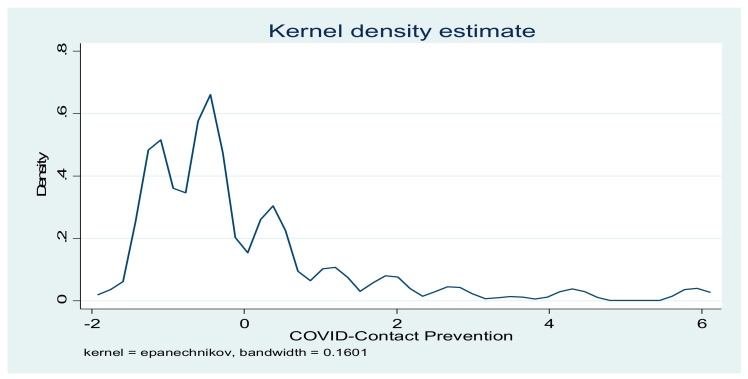
Distribution of COVID-19 contact-prevention indicators.

**Figure 3:**
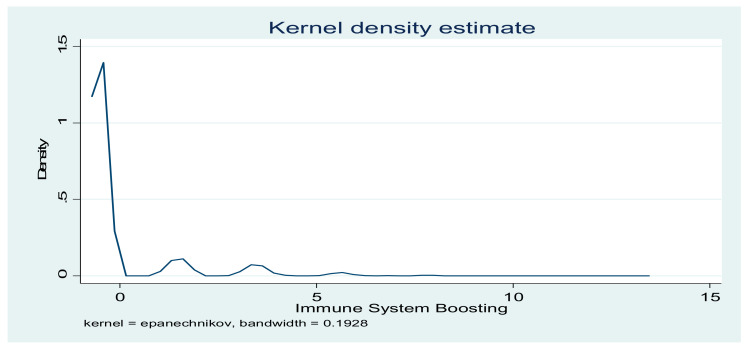
Distribution of COVID-19 immune system-boosting indicators.

The indicators of compliance with COVID-19 preventive practices were computed with PCA. The results in Table 3 show that, for contact-prevention indicators, the first five components explained 70.62% of the total variance. However, for immune system-boosting indicators, 91.09% of the total variance was explained. The distributions of the two indicators are presented in Figure 2 and Figure 3.

Table 4 shows the distribution of respondents’ demographic characteristics across their decisions to either take or reject COVID-19 vaccines. It shows that a willingness to take the vaccines increased from 73.56% during wave 4 to 80.62% during wave 5. However, across the different age groups, the respondents in the 65 years and above age group had the highest proportion (78.31%) of people willing to take vaccines during wave 4, while those between 55 and 64 years had the highest value (83.30%) during wave 5. Furthermore, rural residents had a higher willingness to take vaccines (75.16%) during wave 4, but urban residents recorded a higher value (81.44%) during wave 5. Across gender, male respondents have higher willingness to take vaccines in both waves. Across the different education attainment groups, the results showed that the proportions of the respondents that were willing to take COVID-19 vaccines among those without education (70.78%) and without preprimary education (63.46%) were the lowest during wave 4. Similar results were obtained during wave 5, where a willingness to take vaccines among those without education and preprimary education were 70.69% and 58.97%, respectively. Similarly, across the two periods of data collection, willingness to take COVID-19 vaccines increased, except among the groups without formal education and preprimary education.

### 3.3. Determinants of Contact-Prevention and Immune System-Boosting Compliance

The results in Table 5 reveal the determinants of COVID-19 contact-prevention and immune system-boosting indicators. These results were generated as a prerequisite for analyzing the effect of those indicators on the willingness to take COVID-19 vaccines. The specifications in Equations (2) and (3) therefore present the results in Table 5, after which the residuals were generated for inclusion in the estimation of Equation (4). The results in Table 5 reveal that the models properly fitted the data, going by the statistical significance of the F-statistics (*p* < 0.01). It should also be noted that educational groups were first analyzed with seven individual attainment dummy variables, but some of these groups show a high level of VIF. Therefore, these groups were collapsed into two groups to address the problem of multicollinearity with a single dummy variable. The results showed an absence of multicollinearity with a VIF value of 1.48. The models were also tested for heteroscedasticity using the Breuch-Pagan test. The results of this test showed the presence of heteroscedasticity, with the computed statistics rejecting the null hypothesis of homoscedasticity (*p* < 0.01) in Equations (2) and (3). The equations therefore estimated with proper correction of heteroscedasticity by estimating the parameters with a robust standard error.

The results showed that the instrumental variables—feel anxious and days suffered depression—were highly significant (*p* < 0.01), implying a positive association with the two indicators of compliance with preventive behaviours. Additionally, as the days of feeling nervous increased, indicators of contact prevention significantly decreased (*p* < 0.01). However, as the days feeling lonely increased, the contact-prevention indicator significantly increased (*p* < 0.01), while the immune system-boosting indicator significantly decreased (*p* < 0.05). The parameters on the days of feeling hopeful are indicated with a negative sign in the two models. These results imply that as the days of feeling hopeful increased, indicators of compliance based on contact-avoidance and immune-boosting increased. The results also show that as the days of suffering some physical reactions increased, indicators of contact-prevention and immune-boosting significantly increased and decreased (*p* < 0.05), respectively. Among the demographic variables that were included in the model, urban residence, age and household size did not show statistical significance (*p* > 0.05).

### 3.4. Determinants of Willingness to Take COVID-19 Vaccines

Table 6 presents the results of willingness to take COVID-19 vaccines using panel data. The results showed that the Wald Chi Square statistic is significant (*p* < 0.05). This shows that the model properly fitted the data. The results also indicate the appropriateness of estimating a random effect model, going by statistical significance of the likelihood-ratio test of rho equal to zero (*p* < 0.01). If this test is insignificant, the estimated parameters would be the same as what would be obtained with a standard Probit regression. The value of rho is 0.0867 and this shows that there is a positive contribution by the panel level variance component to the total variance. The model was also tested for parameter stability and robustness using the quadchk command. The results showed parameter stability and robustness, given the very small differences between the results at different integration points.

The results further showed that the parameters of the error terms in Equations (2) and (3), which were included in the model as variables to correct for endogeneity, showed statistical significance (*p* < 0.01). These imply that the two suspected endogenous variables (contact-prevention and immune system-boosting indices) were truly endogenous. Moreover, the first and second hypotheses are to be rejected because the parameters of contact-prevention and immune system-boosting indices are with statistically significant (*p* < 0.01) negative and positive signs, respectively. These results showed that the probability of willingness to take COVID-19 vaccines decreased as the contact-prevention compliance indicator increased. However, the probability of willingness to take COVID-19 vaccines increased as the immune system-boosting compliance increased.

The results further showed that as the number of days of feeling nervous increased, the probability of willingness to take COVID-19 vaccine significantly decreased (*p* < 0.01). An increase in the number of lonely days significantly increased the probability willingness to take COVID-19 vaccines (*p* < 0.01). Out of the demographic variables that were included, only age and attainment of formal education showed statistical significance (*p* < 0.01). The result showed that the probability of a willingness to take COVID-19 vaccines increased as age increased. Individuals that attained some form of formal education had a significantly higher probability of willingness to take COVID-19 vaccines.

## 4. Discussion

The results showed some impressive increases in the proportion of the respondents who were willing to take COVID-19 vaccines between the fourth and fifth waves of the surveys. The recent vaccines’ acceptance rate of 80.62% can, however, be compared to the national coverage of those people that were fully vaccinated, which was 5.4% as of 26 October 2021 [2]. However, the results are showing some positive indications that, as more vaccines are made available, many Kenyans are more likely to get the jab. However, it may take a very long time to attain the required 80% immunization coverage for the attainment of herd immunity [33].

The results further showed the trend of compliance with preventive practices for safeguarding the transmission of COVID-19. Specifically, many of the recommended practices for preventing coronavirus transmission showed increased percentages between the fourth and fifth waves. The results are also different from what was found in some previous studies, where it was reported that compliance with avoiding big groups of people and social distancing declined over time [77,78]. It should be noted that the regular washing of hands, which had been adjudged as one of the best ways for staying protected from the virus, showed an impressive 93.11% compliance in the fifth wave. However, some of the preventive practices, such as hand sanitization and covering the mouth when coughing, were not well utilized.

The results also revealed a very low compliance with those practices that are associated with boosting the immune system. Although there are some controversies on the efficacy of some these food products in enhancing the immune system, some studies have shown some antimicrobial, antioxidant and health-promoting properties of many of them [79,80,81,82]. Specifically, the intake of warm water has been found to enhance the management of fluids in patients with upper respiratory tract infections [79], while garlic possesses some antimicrobial properties [80]. Fruits such as avocadoes and mangoes are excellent sources of roughages, antioxidants and other essential food nutrients.

The results showed a pattern of association between compliance with COVID-19 preventive practices and vaccine hesitancy. The results showed that an increase in compliance with contact-prevention indicators reduced the probability of willingness to get vaccinated. However, an increase in the immune-boosting indicators increased the probability of willingness to get vaccinated. These results are showing some differences in the behaviour of individuals, with respect to their concerns for enhancement of their immune system through diets and avoidance of contacts with the virus. The undertone of the finding is that some individuals have a preference for the COVID-19 contact-prevention practices, thereby creating some reluctancy in getting vaccinated. On the other hand, vaccination was embraced by those who had already taken some actions in ensuring some boosts in their immune systems through the intake of adequate food.

The results further showed that willingness to take the vaccines significantly increased as age increased. Similar findings have been reported [46,82,83,84]. The results showed that the attainment of formal education increased the indicator of compliance with contact-prevention. This finding is contrary to the one that was reported by Padidar et al. [85] but in agreement with that of Valenti and Faraci [46]. In accordance with some previous studies [82,83,84,86,87], willingness to take COVID-19 vaccines increased with educational levels. Additionally, male respondents had lower compliance indicators. Similar findings have been reported in the literature [85]. Contrary to expectation, the respondents that had seen a COVID-19 infected person also had lower compliance indicators.

Some indicators of mental health were included in the model. These variables are essential because of their direct relevance with the functionality of the immune system. The results further showed that anxiety and days with feelings of depression increased the two compliance indicators. The feelings of anxiety can be related to expression of fear in the context of unfolding events during the COVID-19 pandemic [86,87]. Additionally, the number of days with nervous feelings decreased the probability of willingness to take COVID-19 vaccines. The number of days with hopeful feelings decreased the two indicators of prevention compliance and willingness to take vaccines. Feelings of hope may result from evaluation of one’s vulnerability to the pandemic [88,89]. Finally, in line with expectations, the number of days with some physical reactions increased the willingness to take COVID-19 vaccines. This is expected since people with some COVID-19-related symptoms are likely to embrace vaccination because of a high perception of health risk [90].

## 5. Conclusions

The need to understand the effect of compliance with COVID-19 preventive practices on vaccine hesitancy was the major objective of this paper. This is a veritable goal given the dearth of empirical evidence on the form of association that exists between compliance with preventive practices and vaccine hesitancy. This study therefore divided the preventive practices into three indicators, which are contact-prevention compliance, immune system-boosting compliance and vaccination compliance. The findings from this study are pointing at different forms of association between vaccine hesitancy and the prevention compliance indicators. Specifically, there is the need to intensify efforts in promoting the health benefits of some recommended immune boosting practices, such as the intake of vitamin C-rich fruits, garlic, lemon and some fruits in the fight against COVID-19. More importantly, the majority of the respondents are yet to cultivate the habit of consuming these products. Therefore, besides the disease-fighting capabilities of those food products, more compliance with their utilization promises to enhance willingness to take COVID-19 vaccines. In addition, there is the need to properly sensitize the people to the complementary role that exists between compliance with COVID-19 contact-preventing indicators and vaccinations. Such sensitization should clearly highlight the role of immunization in addressing COVID-19 as being distinct from what other preventive practices would accomplish.

It was also found that many of the respondents had suffered from several mental disorders in the form of loneliness, nervousness, depression, hopelessness and anxiety. Essentially, these experiences have different impacts on vaccine hesitancy and compliance with preventive practices. Therefore, there is the need to provide effective platforms for properly managing mental health disorders among people during ongoing pandemics, since experiences such as nervousness and hopelessness reduced their willingness to take COVID-19 vaccines. In relation to some individuals’ demographic characteristics, efforts to promote compliance with preventive practices and reduce vaccine hesitancy should focus on individuals without formal education, males and youths.

## Figures and Tables

**Table 1:** Description of the variables for the regression analyses.

Variable	Mean	Std. Dev.	Min	Max
Dependent variable				
Agree to vaccinate (yes = 1, 0 otherwise)	0.774	-	0	1
Endogenous regressors				
Contact-prevention compliance	1.69 × 10^−8^	1.458	−1.758	5.944
Immune-boosting compliance	−2.12 × 10^−8^	1.370	−0.519	13.270
Exogenous variables				
Days felt depressed in a week	0.428	0.761	0	3
Days felt lonely in a week	0.410	0.752	0	3
Days felt hopeful in a week	1.272	1.274	0	3
Days of physical reactions—nausea, sweating, breathing problem	0.298	0.668	0	3
Urban resident (yes = 1, 0 otherwise)	0.527	-	0	1
Age of the respondent	40.014	13.822	18	98
Gender (male) (yes = 1, 0 otherwise)	0.528	-	0	1
Household size	3.793	2.118	1	17
Formal education (yes = 1, 0 otherwise)	0.975	-	0	1
Know infected person (yes = 1, 0 otherwise)	0.051	-	0	1
Instrumental variables				
Feeling anxious (yes = 1, 0 otherwise)	0.556	-	0	1
Days felt nervous in a week	0.468	0.788	0	3

**Table 2:** Respondents’ compliance with COVID-19 preventive and immune-boosting behaviours.

COVID-19 Preventive/Immune-Boosting Behaviours	Wave 4 (n = 4867)		Wave 5 (n = 5835)		Both Waves (n = 10,702)	
Contact Avoidance Attributes	Frequency	%	Frequency	%	Frequency	%
Hand-washing	4346	89.30	5433	93.11	9779	91.38
Avoiding handshakes	4392	90.24	5638	96.62	10,030	93.72
Wearing masks	3891	79.95	4719	80.87	8610	80.45
Avoiding groups of more than 10 persons	3440	70.68	4766	81.68	8206	76.68
Hand sanitizer	2477	50.89	2995	51.33	5472	51.13
Covering mouth if coughing	1399	28.74	1568	26.87	2967	27.72
Staying at home more	1335	27.43	1376	23.58	2711	25.33
Traveling less	570	11.71	422	7.23	992	9.27
Working less	405	8.32	365	6.26	770	7.19
Stocking up food at home	542	11.14	584	10.01	1126	10.52
Immune-Boosting Attributes						
Drinking tea with lemon	404	8.30	319	5.47	723	6.76
Drinking warm water	404	8.30	600	10.28	1004	9.38
Taking vitamin C rich fruits	134	2.75	120	2.06	254	2.37
Eating lemons, garlic, avocadoes, mangoes	144	2.96	460	7.88	604	5.64
Eating alkaline foods	44	0.90	60	1.03	104	0.97
Taking bicarbonate	88	1.81	34	0.58	122	1.14

**Table 3:** Contributions of each component to total variance.

	COVID-19 Contact-Prevention Indicator			Immune System-Boosting Indicator		
Component	Eigenvalue	Proportion	Cumulative	Eigenvalue	Proportion	Cumulative
Comp1	2.03128	0.2257	0.2257	1.87751	0.3129	0.3129
Comp2	1.51467	0.1683	0.3940	1.07646	0.1794	0.4923
Comp3	0.990668	0.1101	0.5041	0.950245	0.1584	0.6507
Comp4	0.928001	0.1031	0.6072	0.858807	0.1431	0.7938
Comp5	0.891516	0.0991	0.7062	0.702411	0.1171	0.9109
Comp6	0.730758	0.0812	0.7874	0.534568		1.0000
Comp7	0.662303	0.0736	0.8610			
Comp8	0.644016	0.0716	0.9326			
Comp9	0.606791	0.0674	1.0000			

**Table 4:** Distribution of respondents’ demographic characteristics across willingness to take COVID-19 vaccines.

	4th Wave			5th Wave			Both Waves		
Variables	Freq	% Willingness	Total	Freq	% Willingness	Total	Freq	% Willingness	Total
Age									
<25	335	71.89	466	464	77.33	600	799	74.95	1066
25<35	1047	71.13	1472	1452	79.00	1838	2499	75.50	3310
35<45	957	72.89	1313	1256	82.41	1524	2213	78.00	2837
45<55	632	77.83	812	774	81.39	951	1406	79.75	1763
55<65	349	73.94	472	454	83.30	545	803	78.96	1017
65+	260	78.31	332	304	80.64	377	564	79.55	709
Sector									
Rural	1725	75.16	2295	2203	79.70	2764	3928	77.64	5059
Urban	1855	72.12	2572	2501	81.44	3071	4356	77.19	5643
Gender									
Female	1687	73.06	2309	2197	80.04	2745	3884	76.85	5054
Male	1893	74.00	2558	2507	81.13	3090	4400	77.90	5648
Education									
None	109	70.78	154	82	70.69	116	191	70.74	270
Preprimary	99	63.46	156	115	58.97	195	214	60.97	351
Primary	1286	72.53	1773	1387	76.76	1807	2673	74.66	3580
Post primary/Vocational	23	79.31	29	63	80.77	78	86	80.37	107
Secondary	1499	74.95	2000	2288	83.08	2754	3787	79.66	4754
College	426	75.80	562	640	86.37	741	1066	81.81	1303
Undergraduate	119	70.83	168	116	89.92	129	235	79.12	297
Postgraduate	19	76.00	25	13	86.67	15	32	80.00	40
Total	3580	73.56	4867	4704	80.62	5835	8284	77.41	10,702

**Table 5:** Determinant of indicators of compliance with COVID-19 preventive practices.

	COVID-19 Contact-Prevention Model			Immune System-Boosting Model		
Variables	Coef.	Robust Std Error	t Stat	Coef.	Robust Std Error	t Stat
Feel Anxious	0.6949034 ***	0.0267955	25.93	0.6239946 ***	0.0253449	24.62
Days depressed	0.4954908 ***	0.0343624	14.42	0.3213363 ***	0.0313333	10.26
Days nervous	−0.2574125 ***	0.0293624	−8.77	−00.0349017	0.0301548	−1.16
Days felt lonely	0.2634849 ***	0.0274009	9.62	−00.0553346 **	0.0259778	−2.13
Days felt hopeful	−0.1187308 ***	0.0108604	−10.93	−00.0488207 ***	0.0102056	−4.78
Days of physical reactions	0.1869977 ***	0.0378865	4.94	−00.071272 **	0.0307028	−2.32
Urban resident	−0.0094086	0.0261114	−0.36	0.0006897	0.0256915	0.03
Age	−0.0000723	0.0009683	−0.07	0.0000127	0.0009425	0.01
Gender (male)	−0.081161 ***	0.0259716	−3.12	−00.0447487	0.0256916	−1.74
Household size	−0.0020518	0.0061486	−0.33	−00.0082851	0.0061396	−1.35
Formal education	0.1381852	0.079267	1.74	0.0132153	0.0895285	0.15
Know infected person	−0.3011192 ***	0.0571598	−5.27	−0.1179174	0.0638923	−1.85
Constant	−0.5513398 ***	0.0996041	−5.54	−0.314684 ***	0.1058094	−2.97
Number of observations	10,702			10,702		
F(12, 10,689)	128.21 ***			67.44 ***		
R-squared	0.1640			0.0711		
Variance Inflation Factor (VIF)	1.48			1.48		

Note: ***—statistically significant at 1% level; **—statistically significant at 5% level.

**Table 6:** Results of random-effects endogenous probit model.

Variables	Coef.	Standard Error	Z Statistics
Contact-prevention compliance	−1.610935 ***	0.2495848	−6.45
Immune-boosting compliance	203449 ***	0.305411	6.66
Days felt nervous in a week	−0.3476006 ***	0.0545842	−6.37
Days felt lonely in a week	0.4853005 ***	0.0937532	5.18
Days felt hopeful in a week	−0.0357433	0.019011	−1.88
Days of physical reactions—nausea, sweating, breathing problems	0.1555594	0.0807415	1.93
Urban resident	−0.0438994	0.0298572	−1.47
Age of the respondent	0.0045713 ***	0.0011037	4.14
Gender (male}	0.0179088	0.0301979	0.59
Household size	−0.0113539	0.0073404	−1.55
Formal education	0.4981303 ***	0.0952476	5.23
Know infected person	−0.0993262	0.0786707	−1.26
Error term 1	1.502961 ***	0.2493549	6.03
Error term 2	−1.922836 ***	0.305118	−6.30
Constant	0.1769253	0.1235355	1.43
lnsig2u	−2.35439 ***	0.3840399	
Sigma_u	0.3081418 ***	0.0591694	
Rho	0.0867174 ***	0.030415	
Number of observations	10,702		
Integration points	12		
Wald chi2(14)	505.46 ***		
Likelihood-ratio test of rho = 0	8.08 ***		

Note: ***—Statistically significant at 1% level.

## Data Availability

The data used for this study are in the public domain. The data are available at: https://microdata.worldbank.org/index.php/catalog/3774 (accessed on 15 August 2021).
